# Transvesical Laparoscopic Mesh Excision After Tension-Free Vaginal Tape

**DOI:** 10.1155/crog/8824103

**Published:** 2025-06-12

**Authors:** Camille Farache, Peter Fehr

**Affiliations:** Department of Gynecology, Cantonal hospital Graubünden, Chur, Switzerland

**Keywords:** bladder erosion, tension-free vaginal tape, transvesical laparoscopy

## Abstract

Tension-free vaginal tape has been the gold standard for the treatment of stress urinary incontinence for over 20 years. However, rare complications like intravesical tape erosion can significantly reduce quality of life, requiring surgical removal. This procedure must preserve continence and can be challenging. We report the case of a 53-year-old woman who presented with recurrent urinary tract infections and dysuria 7 years after a TVT procedure. Imaging revealed a bladder stone attached to the eroded sling at the bladder neck, inaccessible via standard cystoscopy. A transvesical laparoscopic approach was used to successfully remove the intravesical portion of the tape. The patient recovered well and remained symptom-free and continent. Various techniques are used to perform intravesical tape resection. Transvesical laparoscopy offers excellent visualization and precise excision and minimizes recurrence risks for stress urinary incontinence. It is a safe, effective, and minimally invasive option for removing eroded tension-free vaginal tape, especially in difficult-to-access areas like the bladder neck.

## 1. Introduction

Stress urinary incontinence (SUI) affects 10%–20% of women in the general population [1]. The tension-free vaginal tape (TVT) was developed in the 90s to enable an ambulatory and minimally invasive management for the treatment of this pathology [2]. The use of TVT in the correction of SUI has become widespread, with cure rates exceeding 85% [3]. Postoperative complications are rare but varied. One potential complication is the presence of intravesical tape, either due to intraoperative vesical transfixion or due to secondary intravesical erosion, which can occur several years after the operation [4]. The intravesical tape may cause symptoms of overactive bladder and recurrent infections. Removal of the eroded tape is then required. Different surgical approaches are possible, including transvaginal, endoscopic, and open abdominal methods. Here, we present a transvesical laparoscopic approach to treat bladder erosion.

## 2. Case Report

**Figure 1:**
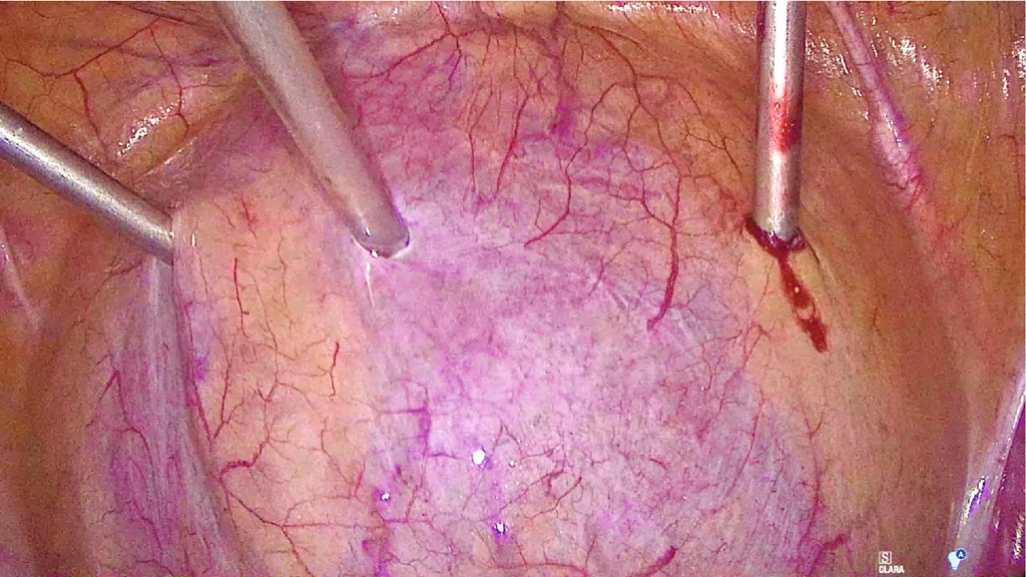
Transvesical 3-mm trocar placement during the procedure.

**Figure 2:**
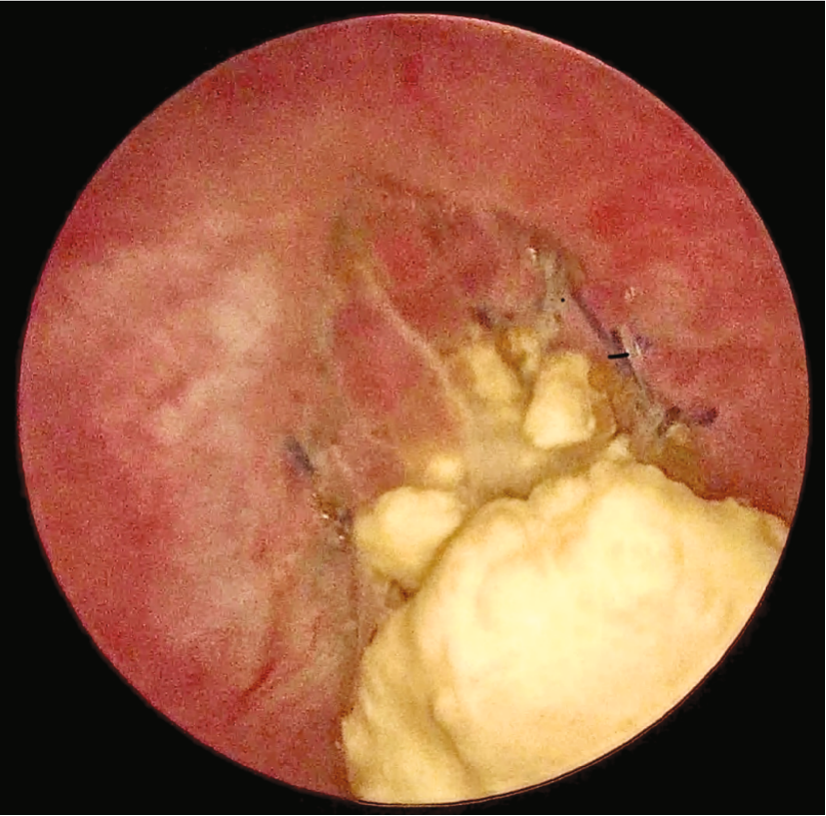
Intravesical tension-free vaginal tape with the stone.

**Figure 3:**
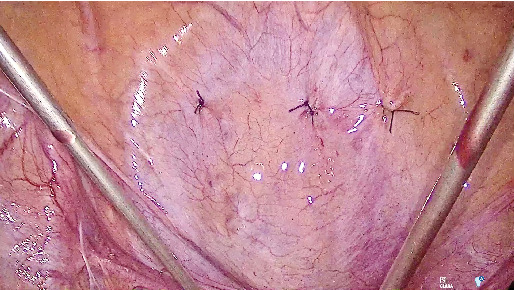
Bladder suture after trocar removal.

A 53-year-old woman with SUI underwent a TVT procedure 7 years ago. She recently reported recurrent urinary tract infections and persistent dysuria. Ultrasound and flexible cystoscopy objectified an intravesical stone attached to the intravesical sling in the bladder neck. She was initially referred to urology for cystoscopic resection of the eroded tape, but the erosion was not visible with a standard cystoscope due to its location in the bladder neck. Therefore, we decided to perform a transvesical laparoscopy. A standard laparoscopy was first performed with an umbilical incision and a 10-mm optic. Examination of the abdominal cavity revealed no abnormalities. Three skin incisions were made 2 cm above the pubic symphysis. After filling the bladder with 300 mL of saline, we inserted three 3-mm trocars through the abdominal wall into the bladder (Figure 1). Through these trocars, we introduced a 3-mm optic and instruments into the bladder. For better visibility, the saline was aspirated and carbon dioxide was insufflated into the bladder. The intravesical tape was dissected and excised using forceps and scissors (Figure 2) and then removed through the urethra. At the end of the procedure, the trocar incisions in the bladder were sutured (Figure 3). The patient was discharged with a Foley catheter on postoperative Day 3, which was removed 7 days later. Fifteen months after the procedure, the patient was fully continent and symptom-free.

## 3. Discussion

Among patients undergoing TVT for SUI, 9.8% experienced complications related to the procedure [5]. Complications like bladder erosions are particularly challenging to manage. Various techniques for removing intravesical slings are described in the literature [6–8], but there is no consensus on the preferred method. Laparoscopic excision of intravesical mesh requires opening the bladder and a full-thickness resection of the bladder wall around the area of the erosion [9]. This means an extensive dissection and the resection of a larger portion of the sling, which increases the risk of SUI recurrence [10]. The transurethral approach does not allow the use of multiple instruments and was not possible in our case due to the proximity of the erosion to the urethra. Indeed, the erosion in the bladder neck was not visible with a standard cystoscope. On the other hand, the transvesical laparoscopic technique is easy to perform, provides good visualization of the eroded tape, and allows the use of either standard or sophisticated laparoscopic instruments. It limits the risk of SUI recurrence by limiting resection to the intravesical part of the tape [11–13].

## 4. Conclusion

Transvesical laparoscopic removal of eroded materials in the bladder is a safe, effective, reproducible, and minimally invasive technique. This method is especially appropriate for erosions in the bladder neck, which are difficult to visualize by cystoscopy.

## Data Availability

The authors confirm that the data supporting the findings of this study are available within the article. Unpublished data are in the patient's medical file and available on request from the authors.
